# Transient increase of interleukin-1β after prolonged febrile seizures promotes adult epileptogenesis through long-lasting upregulating endocannabinoid signaling

**DOI:** 10.1038/srep21931

**Published:** 2016-02-23

**Authors:** Bo Feng, Yangshun Tang, Bin Chen, Cenglin Xu, Yi Wang, Yunjian Dai, Dengchang Wu, Junmin Zhu, Shuang Wang, Yudong Zhou, Liyun Shi, Weiwei Hu, Xia Zhang, Zhong Chen

**Affiliations:** 1 Department of Pharmacology, Key Laboratory of Medical Neurobiology of the Ministry of Health of China, School of Medicine, Zhejiang University, 310058, China; 2 Department of Neurology, Collaborative Innovation Center for Diagnosis and Treatment of Infectious Diseases, First Affiliated Hospital, Zhejiang University, China; 3 Epilepsy Center, Department of Neurology, Second Affiliated Hospital, Zhejiang University, China; 4 Department of Basic Medical Science, Hangzhou Normal University, Hangzhou, China; 5 University of Ottawa Institute of Mental Health Research at the Royal, Department of Psychiatry, and Department of Cellular and Molecular Medicine, Ottawa K1Z 7K4, Canada

## Abstract

It remains unclear how infantile febrile seizures (FS) enhance adult seizure susceptibility. Here we showed that the transient increase of interleukin-1β (IL-1β) after prolonged FS promoted adult seizure susceptibility, which was blocked by interleukin-1 receptor antagonist (IL-1Ra) within a critical time window. Postnatal administered IL-1β alone mimicked the effect of FS on adult seizure susceptibility. IL-1R1 knockout mice were not susceptible to adult seizure after prolonged FS or IL-1β treatment. Prolonged FS or early-life IL-1β treatment increased the expression of cannabinoid type 1 receptor (CB1R) for over 50 days, which was blocked by IL-1Ra or was absent in IL-1R1 knockout mice. CB1R antagonist, knockdown and endocannabinoid synthesis inhibitor abolished FS or IL-1β-enhanced seizure susceptibility. Thus, this work identifies a pathogenic role of postnatal IL-1β/IL-1R1 pathway and subsequent prolonged prominent increase of endocannabinoid signaling in adult seizure susceptibility following prolonged FS, and highlights IL-1R1 as a potential therapeutic target for preventing the development of epilepsy after infantile FS.

Febrile seizures (FS) are the most common (with a prevalence of 2–14%) convulsive events affecting infants between 6 months and 5 years of age^1^,^2^. Although FS are benign in most instances, 30–70% of them are subsequently associated with adult temporal lobe epilepsy (TLE)^3^. Animal studies also have shown that FS rats displayed enhanced hippocampal excitability throughout life^4^, higher risk of adult epilepsy and lower seizure threshold^5^,^6^, which were influenced by the duration of the seizures 6. Still now, however, rarely is known about how infantile FS develop into adult epileptic state, which hampers early diagnosis and prevention. Though traditional anticonvulsant drugs, such as phenobarbital, valproic acid and phenytoin, have been used to treat FS patients, no effective drugs are identified to reduce the risk of adult epileptogenesis^7^,^8^. Therefore, more efforts are needed to elucidate the mechanisms underlying epileptogenesis after FS and further explore new therapeutic drugs.

Seizure activities in clinical cases and in experimental epilepsy models induce rapidly release of proinflammtory molecules in the brain where seizures are generated and spread^9^,^10^. Recently, it has been shown that the interleukin-1β (IL-1β), one of the most important proinflammatory cytokines, is also involved in FS. It is increased after FS^6^ and patients with polymorphisms of IL-1β at position −511 and in exon 5 are more susceptible to develop FS^11^. Moreover, application of high dose of IL-1β results in seizures in immature wild type mice while IL-1R1 knockout mice display higher seizure threshold under hyperthermia environment^12^, suggesting the contribution of IL-1β signaling in the generation of FS. However, all these studies mainly focus on the generation of FS, and whether the increased IL-1β after FS plays an essential role in subsequent epileptogenesis is still not clear.

Therefore, a series of experiments were conducted to investigate the role of IL-1β in epileptogenesis after infantile FS and the underlying mechanism. We found that prolonged, but not simple FS, transiently elevated IL-1β expression in the hippocampus in rodent pups. This elevation produced a prolonged prominent upregulation of cannabinoid type 1 receptor (CB1R) signaling until adulthood, leading to enhanced seizure susceptibility.

## Result

### IL-1β is sufficient to promote adult seizure susceptibility after prolonged FS

**Figure 1:**
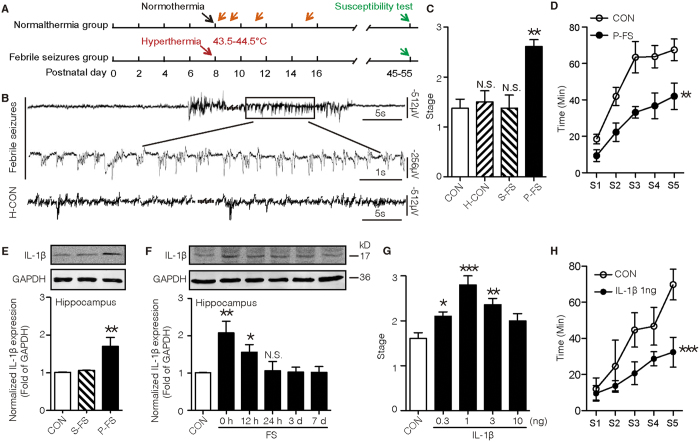
IL-1β promotes seizure susceptibility after prolonged FS. (**A**) Experimental protocols. (**B**) Representative EEG recorded in the hippocampus of an 8-day-old rat pup during prolonged FS. Top two panels: pre-ictal, ictal, post-ictal EEG and the enlargement of the rhythmic discharges. Third panel: hippocampal EEG recorded during hyperthermia in rat pretreated with pentobarbital (H-CON). (**C**) Seizure stages induced by MES in rats experienced simple or prolonged FS (three experiments; n = 7 in control group and n = 8 in other groups). (**D**) Seizure progression induced by KA in rats experienced prolonged FS (three experiments; n = 8 in each group). (**E,F**) Representative Western blots of IL-1β from hippocampi immediately after simple or prolonged FS (**E**), and at different times after prolonged FS (**F**), while GAPDH was used as loading control (Results represent two to three experiments). (**G**) Seizure stages induced by MES in rats treated with different doses of IL-1β on P8 (three experiments; n = 8 for control, n = 7 for other groups). (**H**) seizure progression induced by KA in rats treated with IL-1β on P8 (three experiments; n = 8 in each group; Two-way ANOVA with Bonferroni’s *post hoc* test). One-Way ANOVA with Turkey’s *post-hoc* test. **P < 0.01 and ***P < 0.001 compared to control. Error bars indicate S.E.M. S-FS, simple FS; P-FS, prolonged FS.

Rat pups for FS group (Postnatal day 8, P8; Fig. 1A) were placed under hyperthermia condition (43.5–44.5 °C) to induce seizure. Seizure was confirmed by behavior (see Methods) and hippocampal epileptiform discharges (Fig. 1B), both of which were prevented by pentobarbital. Pups for H-CON group were exposed to hyperthermic environment but pre-treated with pentobarbital. Maximal electrical shock (MES) and kanic acid (KA)-induced seizure models (see Timeline, Fig. 1A) were used to test adult seizure susceptibility. Under 45 mA current stimulation of MES model, rats experienced prolonged FS (P-FS) displayed significantly higher behavioral stages (Fig. 1C), whereas simple FS (S-FS) group and H-CON group (Fig. 1C) displayed similar seizure stage to normothermic group (CON). Similarly, in KA-induced seizure model, prolonged FS-experienced rats displayed faster progression of KA-induced behavioral seizure stage (Fig. 1D) compared with control group. To investigate the role of IL-1β in the generation of enhanced adult seizure susceptibility after prolonged FS, we at first checked the IL-1β expression by Western blot. IL-1β expression increased markedly in the hippocampus immediately after prolonged FS for 12 hours and returned to the baseline 24 hours later, while there was no change in simple FS group (Fig. 1E,F). Moreover, pro-IL-1β was decreased immediately after prolonged FS (Supplementary Fig. 2), suggesting an increased breakdown of immature to the mature form of IL-1β. Furthermore, treatment of 1 or 3 ng IL-1β at P8 elevated MES-induced adult seizure stage (Fig. 1G), and accelerated progression of KA-induced behavioral seizure stage (Fig. 1H), indicating that IL-1β mimics the effect of postnatal prolonged FS on adult seizure susceptibility.

### IL-1β is necessary for the enhanced adult seizure susceptibility after prolonged FS

**Figure 2:**
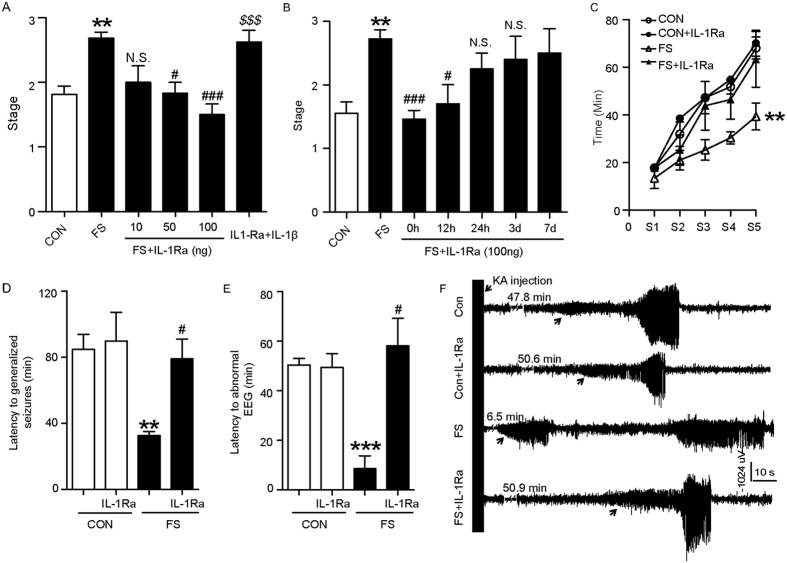
IL-1Ra time-dependently prevents the enhanced seizure susceptibility after prolonged FS. (**A,B**) Seizure stages induced by MES in rats delivered with different doses of IL-1Ra immediately after prolonged FS (**A**, three experiments; n = 8 in FS + IL-1Ra groups, n = 7 in control and FS groups), or treated with 100 ng IL-1Ra at different times after prolonged FS (**B**, three experiments; n = 7–8/group). (**C**) Seizure progression induced by KA with different treatments (n = 10 in control and FS groups, n = 9 in other groups; Two-way ANOVA with Bonferroni’s *post hoc* test). Latency to generalized seizures (**D**) and latency to abnormal EEG (**E**) in rats after KA injection (n = 10 in control and FS groups, n = 9 in other groups). (**F**) Representative EEG tracings from rats following KA injection. The arrow indicates the onset of electrographic seizure.**P < 0.01 and ***P < 0.001 compared to control. ^#^*P* < 0.05, ^###^*P* < 0.001 compared to FS group, ^*$$$*^*P* < 0.001 compared to FS + IL-1Ra group. One-Way ANOVA with Turkey’s *post-hoc* test. Error bars indicate S.E.M. H-CON, hyperthermia without seizures. N.S., not significant.

We then used exogenous recombinant IL1Ra to neutralize endogenous IL-1β to investigate the necessity of IL-1β in the epileptic process. In MES model, exogenous recombinant IL-1Ra at doses of 50 and 100 ng administered immediately after prolonged FS reduced the increased seizure stage (Fig. 2A), which was abrogated by 1 ng IL-1β when administered simultaneously (Fig. 2A). Interestingly, IL-1Ra was also effective when administered within 12 hours following prolonged FS (Fig. 2B) while it was invalid when administered at 24 hours or later (Fig. 2B). In general, 77.8% of prolonged FS rats reach stage 3, while none of the IL-1Ra treated FS rats reached stage 3 (Supplementary Fig. 3). Consistent with that from MES model, administered IL-1Ra lowered the progression of KA-induced behavioral seizure stage (Fig. 2C), prolonged the latency to generalized seizures (Fig. 2D) and to abnormal EEG patterns (Fig. 2E). Figure 2F showed the typical epileptic hippocampal discharges.

**Figure 3:**
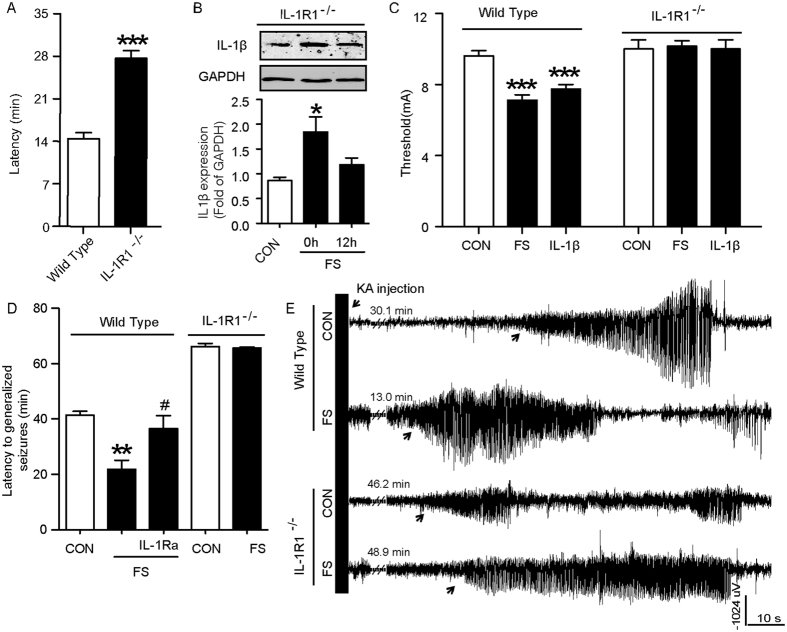
The effect of IL-1β on seizure susceptibility after prolonged FS requires its receptor IL-1R1. (**A**) Latency to the onset of FS, which is a reliable measure of susceptibility, in wild-type and *IL-1R1*^*−/−*^ mice (three experiments; two-tailed Student’s *t-*test, n = 8/group). (**B**) Representative Western blots of IL-1β from hippocampi of *IL-1R1*^*−/−*^ mice, while GAPDH was used as loading control. (**C**) Seizure threshold of MES-induced generalized seizures of wild-type and *IL-1R1*^*−/−*^ mice after FS or IL-1β treatment (two to three experiments; n = 10 for control group, n = 9 for FS and IL-1β in wild-type mice, n = 8 for FS and IL-1β in *IL-1R1*^*−/−*^ mice). Latency to KA-induced generalized seizures in wild-type (**D**) and *IL-1R1*^*−/−*^ mice (**E**) after FS (two-tailed Student’s *t-*test, n = 8/group). (**F**) Representative EEG tracings of mice following KA injection. The arrow indicates the onset of electrographic seizure. **P < 0.01, ***P < 0.001compared to control group. One-Way ANOVA with Turkey’s *post-hoc* test for multiple groups. Error bars indicate S.E.M.

IL-1R1 knockout mice (*IL-1R1*^*−/−*^) were further used to confirm the role of IL-1β/IL-1R1 signaling in epileptogenesis. We first tested the FS generation feature in *IL-1R1*^*−/−*^ mice and found that these pups displayed longer latency to seizure onset compared with age-matched wild-type littermates (Fig. 3A). A subsequent increase of hippocampal IL-1β expression was observed in the *IL-1R1*^*−/−*^ mice (Fig. 3B). When they reached P60, adult seizure susceptibility was tested. In *IL-1R1*^*−/−*^ mice, the threshold to MES-induced seizure was not affected by prolonged FS or IL-1β treatment, while it was reduced in wild-type mice (Fig. 3C). In KA-induced seizure model, the latency to generalized seizures showed no difference between prolonged FS-experienced *IL-1R1*^*−/−*^mice and littermate controls, while it was shortened in wild-type littermates (Fig. 3D). Figure 3E showed KA-induced typical epileptic hippocampal discharges.

### Early life IL-1β treatment induces long-term upregulation of CB1R expression

**Figure 4:**
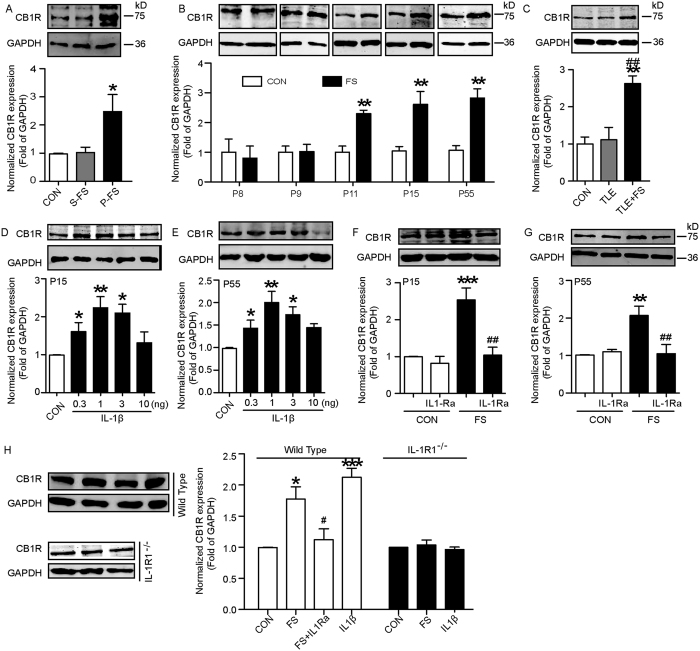
Long-term upregulation of CB1R signaling after prolonged FS. (**A**) Representative Western blots of CB1R protein from hippocampi after simple or prolonged FS. (**B**) Representative Western of CB1R at different times after prolonged FS, while GAPDH was used as loading control. (**C**) Representative Western blots of CB1R from human samples, while GAPDH was used as loading control (three experiments; n = 3 to 5, **P < 0.01 compared to control group, ^##^*P* < 0.01 compared to TLE group). (**D**) and (**E**), Representative Western blots of CB1R protein and the internal control (GAPDH) from hippocampi of rats on P15 (**D**) and P55 (**E**) after the treatment of IL-1β (0.3, 1, 3 and 10 ng). (**F**) and (**G**), Representative Western blots and pooled data for CB1R protein and the internal control (GAPDH) from hippocampi of rats at P15 (**F**) and P55 (**G**) after prolonged FS with or without IL-1Ra treatment. H, Representative Western blots of CB1R protein from hippocampi of wild-type and *IL-1R1*^*−/−*^ mice. One-Way ANOVA with Turkey’s *post-hoc* test for multiple groups. Error bars indicate S.E.M.

Since the epileptic process after prolonged FS is a long-lasting period, further work was needed to elucidate mechanism underlying the proconvulsive effect of the IL-1β/IL-1R1 signaling in enhanced seizure susceptibility. We found an increased expression of CB1R in adult rats (P55), who have experienced prolonged but not simple FS (P55, Fig. 4A). The upregulation of CB1R began at 3 days after prolonged FS and persisted to adulthood (Fig. 4B). Notably, CB1R expression in the epileptogenic foci of TLE+FS patients was 2 times higher than that in TLE patients and non-epilepsy control subjects (Fig. 4C). Since IL-1β (1 ng) alone upregulated CB1R until adulthood (Fig. 4D), it is reasonable to postulate that a link between IL-1β and CB1R may contribute to the enhanced seizure susceptibility. To test the possibility, IL-1Ra and *IL-1R1*^*−/−*^ mice were used to abrogate the upregulation of IL-1β signaling after prolonged FS. We found that IL-1Ra reversed the increase of CB1R expression when administered immediately after prolonged FS (Fig. 4F,G), whereas it was ineffective when administered 24 hours or later (3 days and 7 days) (Supplementary Fig. 4). CB1R expression in *IL-1R1*^*−/−*^ mice was unchanged after prolonged FS or IL-1β treatment, whereas it significantly increased in wild-type littermates (Fig. 4H). Thus, early-life increase of IL-1β after prolonged FS long-lastingly upregulated the CB1R expression.

### The enhanced expression of CB1R is involved in seizure susceptibility

**Figure 5:**
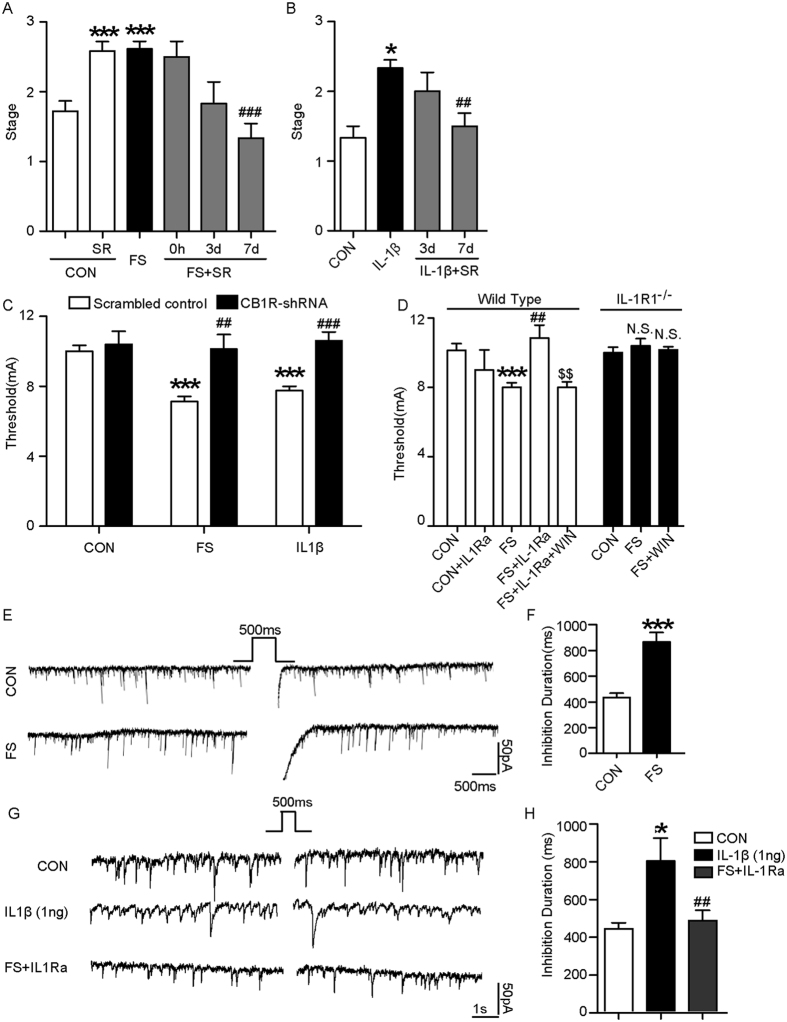
CB1R mediates the effect of IL-1β on seizure susceptibility after prolonged FS. (**A,B**) MES-induced seizure stages of rats treated with SR 141716 A (SR) after FS or IL-1β (three experiments; n = 8 in control group, n = 8 for other groups in A; n = 7 in FS group and n = 6 in other groups). (**C**) Threshold to the onset of MES-induced generalized seizures in mice transducted with CB1R-shRNA or scrambled shRNA lentivirus after prolonged FS or IL-1β injection (three experiments; n = 8/group). (**D**) Seizure threshold to the onset of MES-induced generalized seizures in wild-type and *IL-1R1*^*−/−*^ mice treated with WIN 55,212-2 after prolonged FS or IL-1Ra after prolonged FS (three experiments; wild-type mice: n = 6–8/group). (**E**) Typical traces recorded in the CA1 pyramidal neurons of hippocampal slice from prolonged FS and age-matched control group (two-tailed Student’s *t-*test, n = 22 neurons/group). (**F**) Typical traces recorded in the CA1 pyramidal neurons of hippocampal slice from IL-1β and IL-1Ra treated group (two-tailed Student’s *t-*test, n = 25 neurons/group). *P < 0.05, and ***P < 0.001 compared to control group; ^##^*P* < 0.01, ^###^*P* < 0.001 compared to FS or IL-1β; ^$$^*P* < 0.01 compared to FS + IL-1Ra group. One-Way ANOVA with Turkey’s *post-hoc* test. Error bars indicate S.E.M. N.S., not significant.

To investigate whether CB1R was involved in the enhanced seizure susceptibility following prolonged FS, we used two approaches: CB1R antagonist SR 141716 A and lentiviral mixture (*CB1R-shRNA*) to knock down the CB1R *in vivo*. A single dose of SR 141716A diminished MES-induced seizure stage when administered 7 days after prolonged FS (Fig. 5A). It was ineffective when administered 14 days or 21 days later (Supplementary Fig. 5A). Similarly, it also lowered the MES-induced seizure stage after the early treatment of IL-1β (Fig. 5B). The hippocampal CB1R level was knocked down ~49% by *CB1R-shRNA in vivo* (Supplementary Fig. 5B). Knocking down of CB1R reversed the decrease of threshold to MES-induced seizures after prolonged FS or IL-1β treatment (Fig. 5C), implying that CB1R in the hippocampus was essential in enhanced seizure susceptibility. Furthermore, we activated CB1R with WIN 55,212-2 (a potent CB1R agonist, 0.5 mg/kg, i.p.) 3 days after IL1Ra treatment and found that WIN 55,212-2 reversed the protective effect of IL-1Ra on seizure susceptibility (Fig. 5D). However, WIN 55,212-2 was ineffective in prolonged FS-experienced *IL-1R1*^*−/−*^mice (Fig. 5D; *P* > 0.05).

Activated CB1R on the GABA presynaptic membrane disinhibits (i.e., excite) the postsynaptic neuron, a process called depolarization-induced suppression of inhibition (DSI)^13^,^14^. We tested the CB1-mediated DSI in the hippocampal slice. After 500 ms postsynaptic depolarized stimulation, the duration of DSI in CA1 pyramidal neurons derived from prolonged FS rats (Fig. 5E) and IL-1β-treated rats (Fig. 5F) was significantly longer than that derived from control rats. IL-1Ra reversed the effect of prolonged FS on DSI (Fig. 5F;).

The endocannabinoid system includes CB1R and endocannabinoid and Upregulation of CB1R maybe a compensatory process in response to the change of endocannabinoid tone. We next investigated whether endocannabinoids participate in the enhancement of adult seizure susceptibility. Rats treated with RHC 80267 (an inhibitor of diacylglycerol lipase to endocannbinoid synthesis) 7 days after prolonged FS showed lower seizure stages compared with the vehicle group (Supplementary Fig. 5C). RHC 80267 also reversed the increase of CB1R expression (Supplementary Fig. 5D).

## Discussion

There are three principal findings of our experiments. First, prolonged but not simple FS transiently increases IL-1β expression and enhances adult seizure susceptibility. Second, postnatal treatment of IL-1β enhances adult seizure susceptibility, which is prevented by IL-1Ra in a time-dependent manner. Third, the proconvulsive effect of IL-1β is through long-lasting upregulation of CB1R signaling. These findings shed lights on the role of early life IL-1β/IL-1R1 signaling in the long-term enhanced seizure susceptibility, and suggest that IL-1R1 is a potential target for preventing epileptic event after FS.

Although the polymorphisms of IL-1β have been widely studied, the involvement of the polymorphisms in the susceptibility to FS and TLE is still controversial^15^. So the polymorphisms of IL-1β are not quite useful in predicting FS susceptibility and TLE. Acute changes in cytokine levels (e.g. the interleukins) have been observed in experimental models of adult-onset epilepsy^16^,^17^,^18^, but few studies have explored the role of early-life inflammation-related changes in adult seizures. The coincidence outcome of enhanced seizure susceptibility and increased IL-1β after prolonged but not simple FS gives rise to the hypothesis that the transient change of IL-1β is involved in the long-lasting enhanced seizure susceptibility. Here we provided direct evidence to confirm this hypothesis: 1, IL-1β mimicked the effect of prolonged FS to increase adult seizure susceptibility; 2, IL-1Ra and IL-1R1 deletion blocked the enhancement of adult seizure susceptibility; and 3, the protection of IL-1Ra was reversed by IL-1β. Notably, the proconvulsive effect of IL-1β shows two characteristics: one is that single injection of IL-1β, which mimicked its transient change after FS, showed significant long-lasting effect; the other is that only low doses of IL-1β (e.g., 0.3 and 1 ng) could increase adult seizure susceptibility. Infantile seizures could directly activate microglia for hours^6^, triggering an inflammatory response mediated by cytokines, complement factors and major histocompatibility class factors^18^. Microglia reach their maximal density in the brain during early development^19^ and is involved in synaptic modulation and elimination during the critical period of development in the first few postnatal weeks^20^, which ultimately affect neuronal development. Thus, transient increase of IL-1β and the activated microglia after FS during the critical period of development may also affect synapses formation and show long-lasting influence on neuronal excitability and plasticity. In addition, low concentration of IL-1β exposure potentiates AMPA toxicity on brain slices^21^,^22^, while high concentration of IL-1β induces neuroprotective effects^23^. This may explain our observation that only low dose of IL-1β enhanced adult seizure susceptibility. Our findings indicate that the transient increase of IL-1β indeed participates in infantile prolonged FS-induced adult seizure susceptibility through IL-1R1.

IL-1Ra, as one of the IL-1 family, regulates IL-1 signaling at the receptor level by competing with IL-1β for IL-1RI binding, preventing the formation of a receptor signaling complex and terminating IL-1β-mediated signaling^24^,^25^. In the present study, we found that a single injection of exogenous recombined IL-1Ra could dose-dependently and time-dependently reverse the enhanced adult seizure susceptibility. Interestingly, IL-1Ra was effective only when treated within 24 hours, for the change of IL-1β takes places within 12 hours after FS. Though plasma IL-1Ra/IL-1β ratio was significantly higher in FS patients compared with control children^26^, this endogenous IL-1Ra seems not sufficient to block IL-1β signaling probably because IL-1Ra is produced hours later than IL-1β. Therefore, immediate application of exogenous IL-1Ra is essential to block the IL-1 signaling. Our findings first suggest that control of neuroinflammation in time is an optional and effective strategy for preventing epileptogenesis following early life seizure event.

Previous study demonstrates that mice with IL-1R1 deletion are resistance to the generation of experimental FS^12^, suggesting that IL-1R1 may also serve as a candidate target for controlling FS generation. Besides the antipyretic drugs, traditional anticonvulsant drugs (e.g. phenytoin, diazepam) have been used to treat children with FS^7^,^8^. Unfortunately, these treatments have several limitations. For example, they are ineffective for the later epileptogenesis, and show significant adverse effects on immature brain, including drowsiness, and ataxia. Furthermore, currently available pharmacological treatments of epilepsy mainly focus on seizure suppressing, and none are disease modifying or ‘anti-epileptogenic’^27^. Clinical trials found that Anakinra (Kineret), a recombined IL-1Ra (rIL-RA) approved by the FDA to treatment rheumatoid arthritis, shows no significant adverse events^28^. It also has neuroprotective effects in rodent models of perinatal brain injury^29^,^30^. Given that rIL-RA was approved for the treatment of neonatal-onset multisystem inflammatory disease (NOMID) in 2012, rIL-RA is an ideal candidate drug targeting IL-1R1 to prevent FS generation as well as subsequent epileptogenesis, and translational studies in humans may be feasible.

IL-1β concentration increases transiently after infantile FS, while the subsequent epileptogenesis is a long-term and dynamic process^31^,^32^. How does this transient change of IL-1β modify neuronal excitability in a long-term process? CB1R is a G-protein-coupled receptor that locates at pre-synaptic membrane to mediate retrograde signaling, which tightly controls synaptic plasticity and neuronal excitability^33^,^34^,^35^,^36^. Here we reveal a new role of IL-1β in functional CB1R expression by showing that IL-1β alone can long-lastingly upregulate CB1R over 50 days (Fig. 4). In support, administered IL-1Ra within the critical time window (possible within 24 hours) reversed the prolonged FS-induced CB1R expression, and the upregulated CB1R expression was not observed in FS-experienced or IL-1β-treated *IL-1R1*^*−/−*^ mice. Upregulation of CB1R maybe a compensatory process in response to the change of endocannabinoid tone^34^,^37^. Indeed, endocannabinoid synthesis inhibitor reversed the seizure susceptibility and CB1R expression in our experiment. Additional electrophysiological study and pharmacological manipulations provide more evidence that the infantile IL-1β-induced long-term upregulation of functional endocannabinoid system may be involved in the mechanism underlying adult enhanced seizure susceptibility after prolonged FS. One possible explanation for the long-term modulation of endocannabinoid system is that there may be an epigenetic mechanism controlling CB1R expression. Unfortunately, no DNA methylation of CB1R was observed in the present work (data not shown). In addition to the canonical transcriptional Toll-like receptor pathway, another possible proconvulsive signaling pathway of IL-1β is the non-transcriptional pathway, in which the phosphorylation of the NR2B subunit and increase of intracellular Ca^2+^ in neurons play an essential role^38^,^39^. We speculate that acute and transient increase of IL-1β induces phosphorylation of the NR2B subunit and subsequent neuronal Ca^2+^ influx, resulting in a long-lasting modulation of the endocannabinoid system. Our preliminary data demonstrated that the IL-1β-mediated phosphorylation of the NR2B subunit is involved in the modulation of the endocannabinoid system and the enhanced seizure susceptibility.

In conclusion, the present study demonstrated that acute and transient increase of IL-1β after early life prolonged but not simple FS enhances adult seizure susceptibility through IL-1R1. IL-1Ra prevents this process within a critical time-window. The long-lasting upregulation of encocannabinoid system regulated by IL-1β maybe involved in the underlying mechanism. The present study provides IL-1Ra as new candidate to prevent enhanced seizure susceptibility after infantile prolonged FS.

## Materials and Methods

### Animals

Timed-pregnancy Sprague-Dawley rats and C57BL/6 mice were housed in quiet animal facility cages with a 12-h light/dark cycle. Water and food were available *ad libitum*. The day of birth was considered postnatal day 0 (P0). C57BL/6 backcrossed mice lacking IL-1R1 (Stock number: 003245) were purchased from the Jackson Laboratory. Experiments were carried out between 10:00 and 17:00. All experiments were approved by the Zhejiang University Animal Experimentation Committee and were in complete compliance with the National Institutes of Health Guide for the Care and Use of Laboratory Animals. Efforts were made to minimize the number and suffering of animals.

### Generation of experimental FS

Animals were randomized into control or different treatment groups. The investigator was blinded to the group allocation during experiments. Experimental prolonged FS were induced in rat or mouse pups on postnatal day 8 (P8) as described previously^40^,^41^,^42^. The body temperature of the pups was raised in a warm chamber at 44 ± 0.5 °C for up to 30 min until a seizure was induced. The pups without seizures within 55 min were excluded from further experiments (8/83 of rat pups, 2/28 of wild type mouse pups and 2/22 of *IL-1R1*^*−/−*^ mouse pups). The body weight and core temperature were measured before and after the seizure by a rectal probe (Temperature Control machine, Bowdoinham, ME). Animals were moved out the chamber for 2 min once seizure was evoked. For prolonged FS, pups were returned to the chamber and the procedure was repeated for at least 30 min (prolonged FS). For simple FS, they were returned to the home cage. The behavioral characteristics of the seizures, including sudden movement arrest followed by facial automatisms (chewing), forelimb clonus, and tonic flexion of the body that was often associated with a loss of postural control, were confirmed by EEG recording (Fig. 1B). The latency of hyperthermia-induced seizures showed little variation among different groups. The latency to evoked FS was 23.48 ± 0.44 min in rats (n = 75 total), 17.17 ± 1.12 min in wild type mice (n = 26 total) and 25.66 ± 0.52 min in *IL-1R1*^*−/−*^ mice (n = 20 total). Control animals were littermates of the experimental group and they were all separated from the cages for the same duration and their core temperatures maintained within the normal range.

For hyperthermic controls (H-CON), they experienced the same duration of hyperthermia, but were treated with pentobarbital (30 mg/kg, i.p.; Abbott, North Chicago, IL, USA) 20 min prior to the induction of hyperthermia to block seizures.

### Electroencephalogram (EEG) recording *in vivo*

To test FS generation, pups (P8) were anaesthetized by inhalation of isoflurane (4% for induction and 2% for maintenance) and implanted unilaterally with twisted-wire bipolar electrodes (0.5 mm vertical tip separation) in the hippocampus (rat: AP -1.5, L-1.7, V-3.0, mice: AP -1.3, L-1.5, V-1.6) with a ground electrode placed in the cerebellum. The coordinate was measured in mm from bregma according to the developing brain atlas of Sherwood and Timiras^43^. The electrodes were made of twisted stainless steel Teflon-coated wires (diameter 0.2 mm, A.M. Systems, USA) insulated except at the tip (0.5 mm); the tip separation was about 0.5 mm. The electrodes were connected to a miniature receptacle, which was attached to the skull with dental cement. After 5 hours of recovery, FS was induced and the hippocampal EEG was recorded for 5 min before and throughout the hyperthermia exposure by a digital amplifier (NuAmps, Neuroscan System, USA). The control animals were also electrode implanted for recording.

### Maximal electroshock (MES)-induced seizures

When the animals reached 50–55 days old, electroshocks were delivered using a Rodent Shocker (Hugo Sachs Elektronik, March-Hugstetten, Germany). Electrodes moistened with saline were clipped to the ears. The convulsion patterns were assigned to scores based on the extent of the spread of tonic extension^44^ as follows: 0, absence of forelimb extension; 1, complete forelimb extension without hindlimb extension; 2, complete forelimb extension with partial hindlimb extension; and 3, complete fore- and hindlimb extension (with hindlimbs fully extended parallel to the tail). For rats, in accordance with previous tests in our laboratory^45^, we established the optimal parameters to induce seizures in rats: frequency 50 Hz, shock duration 0.2 s and current 45 mA. The animal was observed for the occurrence of tonic hind limb extension. For mice, the MES threshold was determined by an ‘up-and-down’ method^46^,^47^: the stimulus current intensity began at 5 mA, and the current was lowered 1 mA if the preceding shock caused tonic hindlimb extension or was raised 1 mA if not. The interval between trails is about 5 min.

### Intrahippocampal Kainic acid (KA)-induced seizures

Under sodium pentobarbital anesthesia (35 mg/kg, i.p.; Abbott, North Chicago, IL, USA), rats/mice (~60 days old) were mounted in a stereotaxic apparatus. A cannula was implanted into the right dorsal hippocampus of rats (AP: −3.5 mm, L: −2.4 mm, V: −3.0 mm) and mice (AP: −2.0 mm, L: −1.3 mm, V: −1.6 mm) for KA injection. Electrodes for EEG recording were also implanted in the same area. The electrodes were made of twisted stainless-steel Teflon-coated wires (diameter 0.2 mm; A.M. Systems, USA) insulated except at the tip (0.5 mm); the tip separation was ~0.5 mm. The electrodes were connected to a miniature receptacle. The cannula and miniature receptacle were attached to the skull with dental cement^48^.

To assess the susceptibility to KA-induced seizures, at 7–10 days after implantation surgery, KA (0.5 μg/0.5 μl in normal saline) was injected unilaterally over a period of 5 min through the guide cannula in freely-moving rats/mice^49^. Seizure severity was classified according to Racine^50^: (1) facial movement; (2) head nodding; (3) unilateral forelimb clonus; (4) bilateral forelimb clonus (BFC) and rearing; and (5) BFC with rearing and falling. The EEGs of the hippocampus were recorded 5 min before and 2 hours after KA injection by a digital amplifier (NuAmps, Neuroscan System, USA).

### CB1 receptor RNA interference

Small interfering RNA targeting mouse *CB1R* mRNA (5′-CCC AGA AAA GCA UCA UCA U-3′) was constructed into pLVX-shRNA1 plasmid (Clontech). Lentivirus was produced by co-transfecting a set of plasmids for lentivirus-packing into HEK293T cells according to the manufacturer’s instructions (Clontech). The lentiviruses were collected 48 hours after transfection. For *in vivo* experiments, the lentiviruses at ~2 × 10^9^ transducing units were transfected into mice (2 μl, introhippocampal) at a rate of 0.1 μl/min immediately after prolonged FS. Sham group were injected with viral particles obtained with the same lentiviral vector but containing a scrambled control shRNA sequence (5′-AUG AAC GTG AAU UGC UCA A-3′). The efficacy of CB1R knockdown was assessed by Western blot.

**Western blot** 

The hippocampi were rapidly dissected out and homogenized in RIPA buffer (pH 7.5, in mmol/L; 20 Tris-HCl, 150 NaCl, 1 EDTA, 1% Triton-X100, 0.5% sodium deoxycholate, 1 PMSF, and 10 μg/ml leupeptin). Protein samples (50 or 100 μg samples were loaded for CB1R, pro-IL-1β and IL-1β analysis, respectively) were separated using SDS-polyacrylamide gel electrophoresis and transferred to a nitrocellulose membrane, which was then blocked with 5% skim milk dissolved in PBS (pH 7.4) for 1 h. Then the membrane was incubated with primary antibodies against CB1R (1:500; Abcam), pro-IL-1β and IL-1β (1:200; Abcam) and GAPDH (1:3,000; KangChen) overnight at 4 °C. Secondary antibody against rabbit (IRDye 800-coupled, 1:10,000) was incubated for 2 hours at room temperature. Blots were visualized with the Odyssey infrared imaging system (LI-COR Biosciences) and analyzed with the Odyssey software. The relative optical density was obtained by comparing the measured values with the mean value from the control group.

### Pharmacological agents

The pharmacological agents were administered at the following concentrations *in vivo*: recombinant human IL-1β (Prospec-Tany Techno Gene), 0.3, 1, 3, and 10 ng in 1 μl, i.c.v.^51^,^52^; recombinant human IL1Ra (IL1R antagonist, Prospec-Tany Techno Gene), 2, 20, 50, and 100 ng in 1 μl, i.c.v.^52^; SR 141716 A (CB1R antagonist, Cayman Chemical), 1 and 10 mg/kg, i.p.^53^; WIN 55,212-2 (CB1R agonist, Cayman Chemical), 0.5 mg/kg, i.p.^54^; RHC 80267 (inhibitor of diacylglycerol lipase, Sigma), 1 mg in 2 μl, i.c.v.^55^; KA (Sigma), 0.5 μg/μl, i.c.v. The solvent of SR 141716 A and WIN 55,212-2 was 80% saline +10% ethanol +10% Tween 20.

### Hippocampal slice preparation and drug treatment

Sprague-Dawley rats (P15) were used to prepare hippocampal slices. The hippocampal slices (400 μm) containing hippocampus and parts of adjacent cortex were cut using a vibratome (VT1000S, Leica instruments Ltd., Germany) and equilibrated in an incubation chamber with oxygenated ACSF at 25 °C. The ACSF used in cutting and incubation contained (in mM): 119 NaCl, 2.5 KCl, 2.5 CaCl_2_, 1 NaH_2_PO_4_, 1.3 MgSO_4_, 26.2 NaHCO_3_, and 11 D-glucose (pH 7.4).

Whole-cell patch recordings were obtained in an interface-type chamber at 35 °C, using HEKA EPC-10 amplifier (HEKA Instruments, Germany). Spontaneous IPSCs were recorded in ACSF containing 10 μM D-2-amino-5-phosphovaleric acid (APV; Sigma), 5 μM 6-cyano-7-nitroquinoxaline-2, 3-dione (CNQX; Sigma)^35^. Patch pipettes were pulled from glass capillaries with an outer diameter of 1.5 mm on a two-stage puller (PC-10, Narishige). The resistance between the recording electrode filled with the pipette solution and the reference electrode was 3-5 MΩ. The patch pipette solution for DSI experiments contained (in mM): 140 CsCl, 5 NaCl, 0.2 EGTA, 2 MgATP, 0.3 Na_3_GTP, 5 QX-314, 10 Na_2_HPO_4_ and 10 HEPES (pH 7.2). The depolarizing pulses used to evoke DSI were stepped to 0 mV from the holding potential (usually −60 mV) lasting 500 ms. Off-line analysis was performed using Mini-analysis (SynaptosoftInc, GA, USA).

### Human material

Hippocampal tissues from the patients intractable epilepsy with or without history of FS were confirmed to the following criteria: (i) showing typical epilepsy symptoms and electroencephalographic features, and seizures persisting more than 2 years of medical therapy with three or more kinds of antiepileptic first-line drugs with effective blood drug concentrations; (ii) seizure syndromes fitting the 1981 International Anti-epilepsy Federation classification; (iii) no progressive foci found after computed tomography and magnetic resonance imaging examination; (iv) no other nervous system diseases and no potential inducing causes; and (v) localization-related epileptiform discharges in preoperative evaluation and operative indications. Cortical tissue from temporal lobes of brain trauma was stored as control tissue. Control group criteria: (i) no history of central nervous system disease; and (ii) no structural and functional injury that can induce epilepsy other than trauma. There was no statistical difference in age between the groups. The clinical characteristics were summarized in supplementary Table 1.

Written informed consent was obtained from both epilepsy and control subjects and signed by subjects and legal guardians. The research was approved by the Medical Ethical Committee of Zhejiang University School of Medicine and the methods were carried out in accordance with the approved guideline. Epileptic focus location and operation were monitored by an intraoperative electrode. Abnormal waves disappeared or were markedly reduced in the immediate electrocorticography examination after the resection of foci and tissues with abnormal discharges near the foci.

### Human tissue preparation and western blot analysis

One gram of temporal cortical tissue was dissected from fresh frozen brain sections maintained at −80 °C. Proteins were immunoblotted using antibodies to CB1R. Blots were visualized with the Odyssey infrared imaging system (LI-COR Biosciences) and analyzed with the Odyssey software. The relative optical density was obtained by comparing the measured values with the mean value from the control group.

### Histology

After seizure induction, pups with electrolytic implantation were anesthetized and perfused transcardially with 4% paraformaldehyde. The brains were separated and stored in 4% paraformaldehyde at 4 °C for 24 hours, and then in 30% sucrose for 5–7 days. The brains were sectioned and stained with toluidine blue to examine the electrode sites (Supplementary Fig. 1).

### Statistical analyses

All data were collected and analyzed in a blind fashion. Data are presented as mean ± S.E.M. Comparison between groups was made using *t*-test. One-way ANOVA with Turkey’s *post-hoc* test and Two-way ANOVA with Bonferroni’s *post hoc* test were used for multiple comparisons. *P* < 0.05 was considered statistically significant.

## Additional Information

**How to cite this article**: Feng, B. *et al.* Transient increase of interleukin-1β after prolonged febrile seizures promotes adult epileptogenesis through long-lasting upregulating endocannabinoid signaling. *Sci. Rep.* **6**, 21931; doi: 10.1038/srep21931 (2016).

## Supplementary Material

Supplementary Information
