# Supplementary material for: Transient increase of interleukin-1β after prolonged febrile seizures promotes adult epileptogenesis through long-lasting upregulating endocannabinoid signaling
Source: Sci Rep. 2016 Feb 23;6:21931. doi: 10.1038/srep21931 (PMC4763292; doi:10.1038/srep21931)
Supplement: Supplementary Information [file srep21931-s1.pdf]

Submitted to Scientific Reports

Transient increase of interleukin-1β after prolonged febrile seizures promotes adult epileptogenesis through long-lasting upregulating endocannabinoid signaling

Bo Feng[1,a], Yangshun Tang[1,a], Bin Chen[1], Cenglin Xu[1], Yi Wang[1], Yunjian Dai[1], Dengchang Wu[1,2], Junmin Zhu[3], Shuang Wang[3], Yudong Zhou[1], Liyun Shi[4], Weiwei Hu[1], Xia Zhang[5] and Zhong Chen[1,2,3,*]

Supplementary Table 1. Summary of clinical characteristics

	Gender	Localization	Age of surgery (year)	Age of seizure onset (year)	Duration of epilepsy (year)	Pre-operative seizure frequency last month	Post-operative outcome (Engel's score)
TLE patient							
1#	F	R	27	15	12	4	Ia
2#	F	R	40	37	3	50	Ia
3#	F	L	34	32	2	3	Ia
4#	M	R	22	15	7	1	Ia
5#	F	L	21	16	5	20	II
FS-TLE patient							
7#	F	R	27	15	12	10	Ia
8#	F	R	22	8	14	6	Ia
9#	F	R	41	24	17	1	Ia

TLE: Temporal lobe epilepsy; F: female; M: male; R: right; L: left

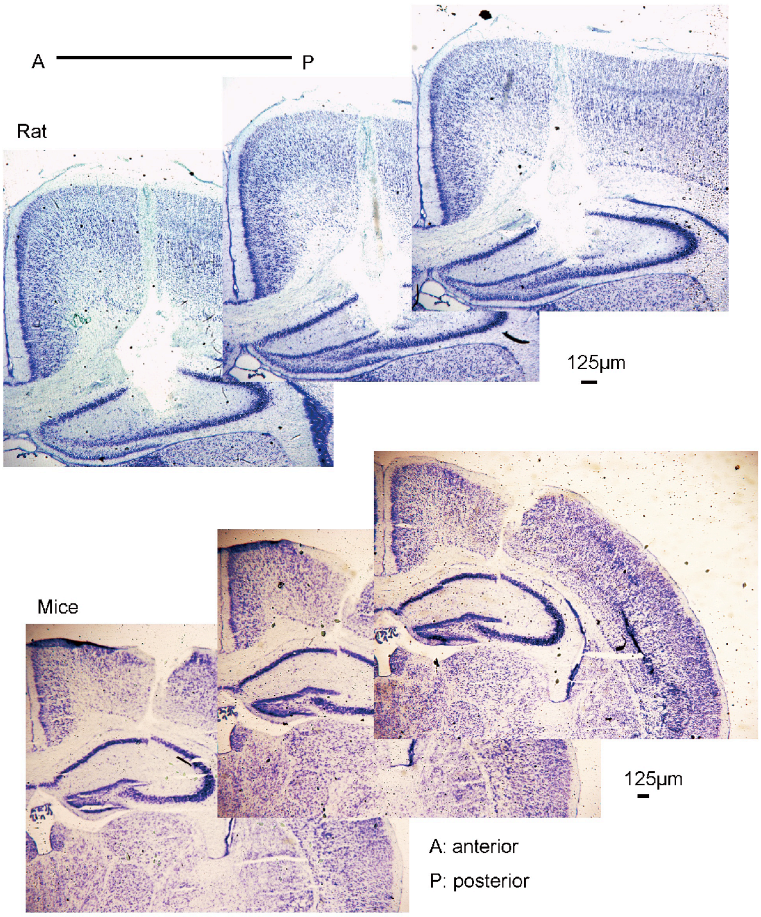

Supplementary Fig.1 Photomicrograph of the coronal section after deep electrode implantation in rat and mice. Bar = 125μm.

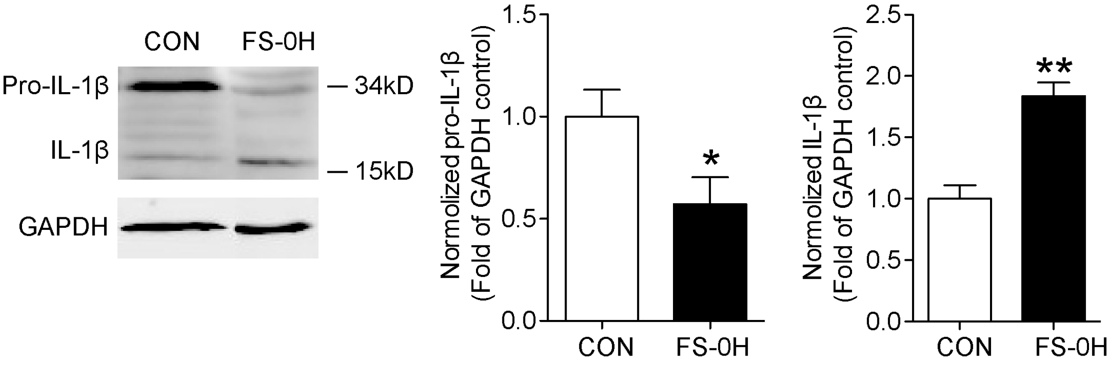

Supplementary Fig.2 Pro-IL-1β decreases immediately after prolonged FS. Representative Western blots of Pro-IL-1β and IL-1β from hippocampi after prolonged FS, while GAPDH was used as loading control.

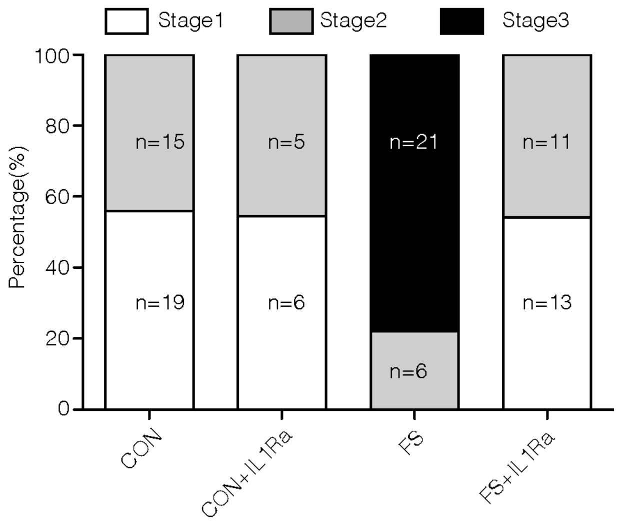

Supplementary Fig.3 Stage position of rats reaching stages 1, 2 and 3 of MES-induced seizures.

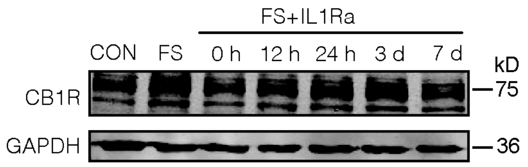

Supplementary Fig.4 Representative Western blots and pooled data for CB1R protein and the internal control (GAPDH) from hippocampi at P15 after prolonged FS with IL-1Ra treatment at different times.

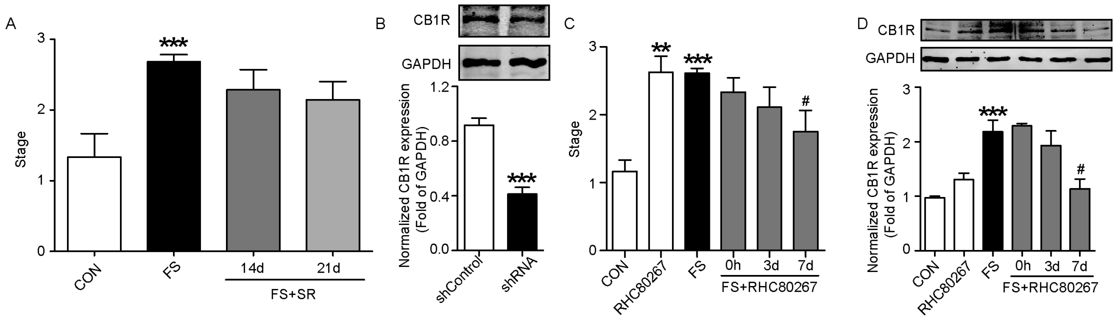

Supplementary Fig.5 A, Seizure stages induced by MES in rats received SR at 14 or 21 days after prolonged FS. B, Representative Western blots and pooled data of CB1R protein and the internal control (GAPDH) from hippocampi with CB1R-shRNA or scrambled shRNA *in vivo*. C, Seizure stages induced by MES with or without RHC 80267 in rats (three experiments; n = 6-8/group. D, Representative Western blots and statistics of CB1R protein from hippocampi in rats treated with RHC80267, 7 days after prolonged FS (three experiments). ***P* < 0.01 and ****P* < 0.001 compared to control group, #*P*<0.05 compared to FS group. One-way ANOVA with Turkey's *post-hoc* test. Error bars indicate S.E.M.
